# Intra-epidemic genome variation in highly pathogenic African swine fever virus (ASFV) from the country of Georgia

**DOI:** 10.1186/s12985-018-1099-z

**Published:** 2018-12-14

**Authors:** Jason Farlow, Marina Donduashvili, Maka Kokhreidze, Adam Kotorashvili, Nino G. Vepkhvadze, Nato Kotaria, Ana Gulbani

**Affiliations:** 1 Laboratory of the Ministry of Agriculture, Tbilisi, Georgia; 2 Richard G. Lugar Center for Public Health Research at the National Center for Disease Control (NCDC), Tbilisi, Georgia; 3 Farlow Scientific Consulting Company, LLC, Lewiston, UT 84320 USA

## Abstract

**Background:**

African swine fever virus (ASFV) causes an acute hemorrhagic infection in suids with a mortality rate of up to 100%. No vaccine is available and the potential for catastrophic disease in Europe remains elevated due to the ongoing ASF epidemic in Russia and Baltic countries. To date, intra-epidemic whole-genome variation for ASFV has not been reported. To provide a more comprehensive baseline for genetic variation early in the ASF outbreak, we sequenced two Georgian ASFV samples, G-2008/1 and G-2008/2, derived from domestic porcine blood collected in 2008.

**Methods:**

Genomic DNA was extracted directly from low-volume ASFV PCR-positive porcine blood samples and subjected to next generation sequencing on the Illumina Miseq platform. De novo and mapped sequence assemblies were performed using CLCBio software. Genomic illustrations, sequence alignments and assembly figures were generated using Geneious v10.2.4. Sequence repeat architecture was analyzed using DNASTAR GeneQuest 14.1.0.

**Results:**

The G-2008/1 and G-2008/2 genomes were distinguished from each other by coding changes in seven genes, including MGF 110-1 L, X69R, MGF 505-10R, EP364R, H233R, E199L, and MGF 360-21R in addition to eight homopolymer tract variations. The 2008/2 genome possessed a novel allele state at a previously undescribed intergenic repeat locus between genes C315R and C147L. The C315R/C147L locus represents the earliest observed variable repeat sequence polymorphism reported among isolates from this epidemic. No sequence variation was observed in conventional ASFV subtyping markers. The two genomes exhibited complete collinearity and identical gene content with the Georgia 2007/1 reference genome. Approximately 56 unique homopolymer A/T-tract variations were identified that were unique to the Georgia 2007/1 genome. In both 2008 genomes, within-sample sequence read heterogeneity was evident at six homopolymeric G/C-tracts confined to the known hypervariable ~ 7 kb region in the left terminal region of the genome.

**Conclusions:**

This is the first intra-epidemic comparative genomic analysis reported for ASFV and provides insight into the intra-epidemic microevolution of ASFV. The genomes reported here, in addition to the G-2007/1 genome, provide an early baseline for future genome-level comparisons and epidemiological tracing efforts.

**Electronic supplementary material:**

The online version of this article (10.1186/s12985-018-1099-z) contains supplementary material, which is available to authorized users.

## Introduction

African Swine Fever Virus (ASFV) is a large, icosahedral, dsDNA virus and sole member of the *Asfarviridae* family [1]. ASFV is the only known DNA arbovirus [2]. ASFV is highly adapted for both sylvatic (enzootic) and domestic transmission cycles and infects wild boars (*Sus scrofa scrofa*), warthogs (*Phacochoerus aethiopicus*), bushpigs (*Potamochoerus*spp.), domestic pigs and *Ornithodoros* ticks [1, 3–5]. Following the 2007 ASF outbreak in the Caucasus, ASFV has been detected in Russia, Ukraine and Baltic countries [6–8].

Conventional genetic subtyping strategies for ASFV utilize tandem-repeat sequences (TRSs) within p72 (B646L), p54 (E183L), the central variable region (CVR) of the B602L gene and an intergenic region between *I73R* and *I329L* [9–14]. More recently, loci within p30 and EP402R (CD2v) have also shown discriminatory utility [15]. Previous studies have identified at least 22 distinct p72 genotypes among East and South African isolates with genotype I representing the major genotype present in West Africa and genotype II predominating in East Africa [15, 16]. Thus far, only p72 genotype II has been detected in eastern Europe and Russia following a putative single introduction of this genotype into Georgia in 2007 [17]. An insertion observed at the *I73R*/*I329L* TRS marker also confirmed allele variation in Russia or elsewhere in the Caucasus prior to the detection of the virus in Baltic countries [7, 8]. While existing ASFV marker-based assays provide genetic discrimination among isolates, higher-resolution genomic comparisons may facilitate more robust epidemiological tracing [18]. Lack of whole genome data for both closely related endemic strains as well as those generated during intra-epidemic transmission remain unavailable. To date, only a single ASFV genome sequence has been reported from the current epidemic (G-2007/1) [19].

We report here a comparative genomic analysis of two ASFV isolates (G-2008/1 and G-2008/2) sequenced directly from PCR-positive porcine blood isolates taken in 2008 during the initial phase of the outbreak in Georgia. These data represent the first intra-epidemic comparative genomic analysis of ASFV, and to our knowledge, represent the first ASFV genome-level sequences derived directly from naturally-infected suids without prior cell culture propagation. These data also provide insight into the wild-type genome microevolution of ASFV and provide an additional baseline for genetic variation early in the ASF outbreak.

## Materials and methods

Genomic DNA was extracted directly from low-volume ASFV PCR-positive porcine blood samples, designated G-2008/1 and G-2008/2, using the DNeasy Blood & Tissue kit (QIAGEN, Hilden, Germany). Village and district-level geographical locations were not available for the isolates. Genomic libraries were quantified using the Qbit (Qbit 2.0 Fluorometer, Life technologies) and sheared using the Covaris M220 focused ultrasonicator (Covaris, Inc.) Genome fragmentation was performed on the BioAnalyzer 2100 (Agilent Technologies, Inc.). Libraries were subsequently modified using the Illumina Paired End Sample Prep Kit and sequenced on the Illumina MiSeq platform (Illumina, Inc.) per manufacturer’s instructions. Raw fastq files for each virus sample were subjected to de novo assembly using CLCBio software (CLC Bio, Aarhus, Denmark). Mapped assemblies were also performed using the previously reported ASFV Georgia 2007/1 sequence (FR682468) as a reference. Variant calling, genome alignments and sequence illustrations were generated using Geneious software version 10.2.4. The C315R/C147L locus from global representatives were aligned using available nucleic acid sequence data from Genbank (Fig. 2c).

## Results

**Fig. 1:**
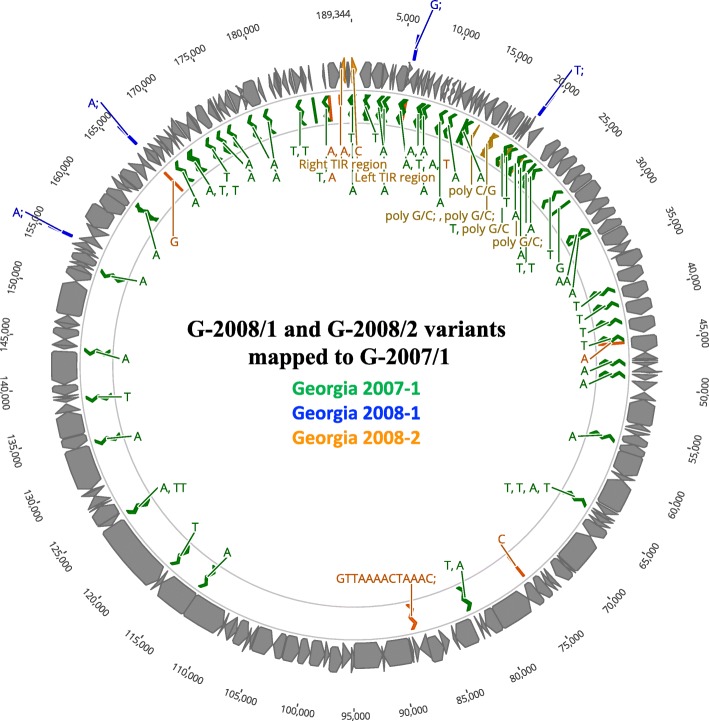
Genomic polymorphisms among Georgian ASFV isolates mapped to the Georgian 2007/1 reference genome. Variants unique to each of the three genomes are designated by separate colors (G-2007/1 = green, G-2008/1 = blue, G-2008/2 = orange)

Illumina deep sequencing recovered near full length draft genomes for the two isolates. Average read coverage obtained for G-2008/2 (118X) was significantly higher than G-2008/1 (8.5X). A 547 bp region spanning the 3′ end of the annotated left terminal inverted repeat (TIR) region and the proximal end of the first open reading frame, multigene family (MGF) protein 360-1 L, was not recovered in the 2008/2 genome, despite significant read depth across the remainder of the genome. The architecture of the ASFV genome and genetic polymorphisms are illustrated in Fig. 1 and Table 1. The two genomes exhibited complete collinearity and identical gene content with the Georgia 2007/1 reference genome. The G-2008/1 and G-2008/2 genomes were distinguished from each other by coding changes in seven genes, including MGF 110-1 L, X69R, MGF 505-10R, EP364R, H233R, E199L, and MGF 360-21R in addition to eight homopolymer tract variations in the left (*N* = 6) and right (*N* = 2) terminal regions (Table 1). Molecular targets used in conventional ASFV subtyping assays, including p72 (B646L), P54 (E183L), CVR (pB602L), and the *I73R*/*I329L* TRS locus exhibited no sequence variation. Genome comparisons also revealed 56 unique homopolymer A/T-tract variations unique to the Georgia 2007/1 reference genome (Table 1).

**Table 1:**
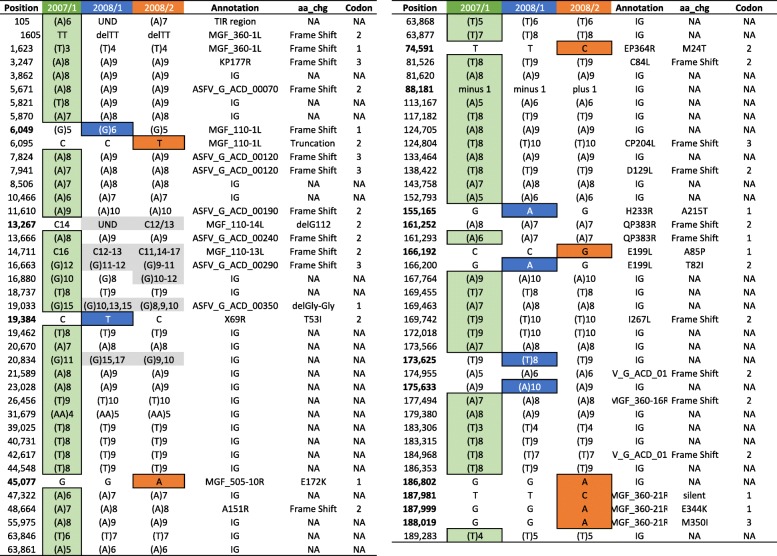
Genome-wide genetic polymorphisms identified between ASFV isolates 2007/1, 2008/1, and 2008/2

**Fig. 2:**
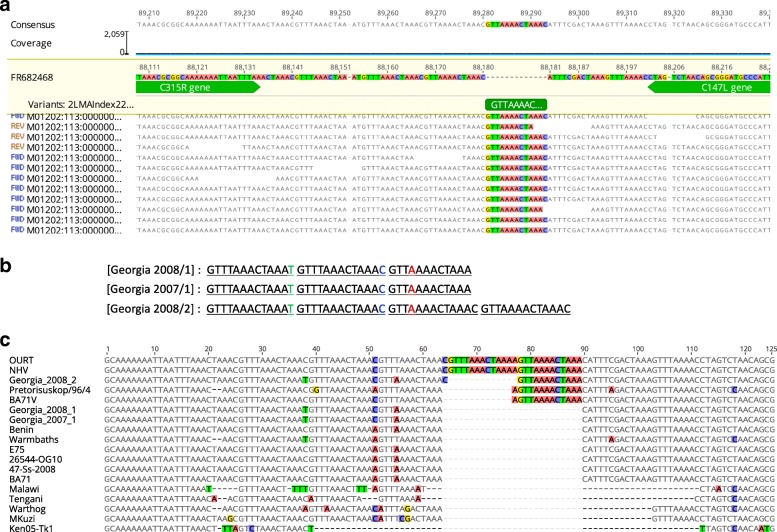
**a** Sequence read mapping of the C315R/C147L locus TR insertion in 2008/2 at the intergenic region between genes C315R (TFIIB-like protein) and C147L (RNApol subunit 6). **b** Repeat units of 2007/1, 2008/1, and 2008/2. **c** Nucleic acid sequence alignment of the C315R/C147L locus among global ASFV representatives. Colored residues indicating variable SNPs among global strains in C are also highlighted in section B

A novel intergenic sequence insertion (GTTAAAACTAAAC) was observed in the G-2008/2 genome between genes C315R (TFIIB-like protein) and C147L (RNApol subunit 6) (Fig. 2a, b, and c). The short tandem repeat array (Fig. 2b) forms a portion of a larger indirect repeat structure illustrated in Additional File 2 (Additional file 1: Figure S1D and E). This locus displays significant copy unit diversity among global ASFV strains (Fig. 2c). The length of the intergenic region among global strains ranged from 20 bp (Ken05-Tk1) to 93 bp (OURT and NHV) (Fig. 2b). The limits of the indel boundaries extended to the terminal end of C147L gene.

Within-sample sequence read heterogeneity occurred at six homopolymeric G/C-tracts confined to a ~ 7 kb region in the LTR (13,267-20,834) (Table 1, Fig. 1). This region is illustrated in further detail in Additional File 1 (Additional file 1: Figure S1A-C). These loci were found to possess approximately 10 or more residues and represented the only G/C homopolymer tracts of this length within the genome. Variable sites included a homopolymeric C-tract in MGF_110_13L, two homopolymeric G-tracts in genes ACD_00290 and ACD_00350, respectively, and two intergenic homopolymeric tracts (Table 1). This variability may represent sequencing artifact generated during library amplification or derives from within-host genome variation during natural infection.

Genome sequences of the Georgia 2008/1 and Georgia 2008/2 strains were deposited in GenBank under accession numbers MH910495 and MH910496, respectively.

## Discussion

The genomes reported here provide additional references that may inform epidemiological tracing of related intra-epidemic isolates and provides insight into viral microevolution during acute wild-type infection. The novel allele at the C315R/C147L locus currently represents the earliest observed repeat variation among isolates derived from the current epidemic lineage. ASFV genomes exhibit elevated diversity in the multi-gene family (MGF) genes located in the left and right variable regions (LVR and RVR, respectively) [19–23]. Approximately 11 of the fifteen total SNPs that differentiated the two 2008 genomes were non-synonymous. The remaining four SNPs represent intergenic homopolymer tract variation. Additional genomic data from this epidemic lineage may allow an expanded assessment of constraints on synonymous variation.

### Homopolymer tract variation

Resolution of correct homopolymer tract allele states is warranted due to their ability to generate frameshift mutations, alter amino acid repeat number and protein structure, produce gene fusions, and alter putative promoter and termination signals. Substitution mutations and homopolymer tract variations among the Georgian ASFV genomes were elevated in the terminal regions of the genome, particularly in the LVR (Fig. 1). It is unclear whether the homopolymer tract diversity present in the Georgia 2007/1 isolate genome reflects the original wild-type sequence, sequencing error, or mutations arising in cell culture prior to harvesting. Homopolymer tract sequences in the Georgia 2007/1 genome were reported to have been validated using Sanger sequencing [19]. The G-2008/1 and G-2008/2 genomes were sequenced directly from porcine blood samples. In contrast, the Georgia 2007/1 isolate was obtained from infected porcine organ tissue, cultured, and harvested from primary porcine bone marrow cells [19].

Under a scenario where the 2007/1 genome represents the ancestral sequence, the subsequent emergence and maintenance of homopolymeric A/T variations fixed in the G-2008/1 and G-2008/2 genomes would suggest a rapid and stable adaptation to the local host population. Previously published whole genome data gathered following extensive in vitro passage of individual strains revealed only minimal poly A/T tract variability. Whole genome comparisons of the Vero cell culture-adapted BA71V virus and the highly virulent BA71 parent strain revealed only five variable homopolymeric A/T loci and no G-tract polymorphisms [24]. Furthermore, no homopolymeric tract variations were reported in Vero-adapted Georgia 2007/1 strains (ASFV-G passages 30, 60, 80, and 110) although these genomes are not publicly available for independent verification [25]. Compared to such in vitro data, the extent of homopolymeric A/T-tract variability observed between the G-2007/1 genome and G-2008/1 and G-2008/2 appears pronounced, although the context of genome evolution may be expected to differ between published in vitro studies and wild-type genome variation reported here. We found that select homopolymeric A/T-tract loci unique to the 2007/1 genome in our analysis also displayed variation in previous studies, such as the terminal poly-T tract the CP204L gene and the homopolymer tract responsible for fusion of MGF 110-13 L homologs in L60, E75, and NHV28. Homopolymer-based variability has also previously been described in 14 additional ASFV proteins, including p54 and E183L [26, 27]. Homopolymeric tracts that displayed notable tract length variability in other strains, such as that within the M1249 L gene of strains L60 and E7528 showed single-allele state in the three Georgian ASFV genomes here.

### Molecular marker stability

Cell culture propagation is frequently performed prior to ASFV genome sequencing. The genetic stability of ASFV epi-markers in traditional ASFV in vitro cell culture systems remains understudied. Limiting errors in phylogenetic inference, such as those arising from homoplasious variation and evolutionary selection are a crucial component of molecular assay design strategy. The extent of in vitro passage or natural transmission events needed to mediate homoplasious variation in current ASFV molecular markers remains unknown and warrants further investigation. We find previous data indicate in vitro propagation may alter genetic loci currently used for ASFV subtyping. Our analysis of genome sequence data generated in a previous study [24] describing adaption of strain BA71 to Vero cells revealed that the adapted strain BA71V uniquely possessed a new CVR (pB602L) allele that is identical to that of the Portuguese strain Por 63 (Supp. 1F). Homoplasious variation was also found to occur at the CVR (B602L) marker in select global strains during replication in pig macrophages and Vero cells [28].

In addition to their use in ASFV subtyping, tandem repeat (TR) markers are also used for dissecting molecular variation in other dsDNA viruses including Hepatitis C virus [29], Adenoviruses [30], human Cytomegaloviruses [31], and Herpesviruses [32–34]. The approach used by Deback et al. is similar to that commonly used in developing microbial variable-number tandem-repeat (VNTR) assays. This approach includes selection of candidate TR loci that are first informed by comparative genome analyses and subsequently tested for genetic stability in vitro [32]. This approach represents a viable strategy for testing existing ASFV marker stability as well as future markers relevant for tracing current intra-epidemic ASFV isolates. Cataloguing genome-wide variation among a longitudinal set of geographically diverse isolates from the current epidemic would facilitate the identification of new more epidemiologically-informative marker loci. The genomes reported here, in addition to the G-2007/1 genome, provide an early baseline for such efforts.

Variability in the genetic architecture of the genomic region spanning the six hypervariable G/C-tract loci in our study has been reported previously [35, 36]. The repeat tracts are positioned at the core of homopolymeric A + T rich regions within and adjacent to select MGF 110 and MGF 300 genes that together form diverse larger internal repeat sequences in the LVR (38). These regions were previously shown to possess architectural similarity to eukaryotic scaffold-associated regions (SARs) and satellite III repeats of *Drosophila* [36]. Whether the sequence read heterogeneity at these loci observed in our data represent artifact or a natural mutant spectrum is unclear. Lack of sample volume prevented further molecular validation. Each homopolymeric locus uniquely contained ≥10 Gs or Cs and was confined to the same 7 kb region in the LVR and is illustrated in Additional file 1: Figure S1A. All remaining homopolymeric G/C-tracts in the G-2008/1 and G-2008/2 genomes possessed less than 10 residues and displayed no within-sample copy number variation. Homopolymer A/T tract loci in the genome, by comparison, exhibited no detectable intra-sample variation. In contrast to the G-2007/1 genome, the G-2008/1 and G-2008/2 genomes exhibited only two homopolymeric A/T-tracts that differed and displayed no within-sample heterogeneity in their sequence read populations. The basis for the occurrence of within sample homopolymer tract variation confined only to this region of the genome in both isolates remains unknown.

### Wild-type genomic homogeneity

To date, this study is the first analysis of intra-epidemic ASFV genome sequences and provides insight into genome stability during wild-type infection. DNA viruses generally display low mutation rates largely due their encoded high fidelity DNA proofreading repair enzymes. ASFV genomes uniquely encode the only known X-type polymerase (X Pol) and a DNA ligase that each exhibit low fidelity [37, 38]. The in vitro fidelity of the ASFV DNA ligase is currently the lowest known. The genetic and phenotypic basis for the extreme antigenic diversity displayed by ASFV strains has not been determined [39, 40]. Elevated antigenic diversity in field isolates has been speculated to arise from the activity of the X Pol/DNA ligase. Previous authors have hypothesized that the Pol X/ligase system functions as a strategic DNA mutator in the base excision repair (BER) pathway where rapid genetic drift occurs due to elevated DNA repair-based error [37, 41, 42]. Conflicting data for Pol X fidelity have been reported [37, 41, 43]. It has been speculated that the incongruous reports resulted from enzyme redox state differences between the published studies [44], wherein greater fidelity is exhibited by the reduced form of the enzyme [45]. We speculate realization of the DNA mutator model proposed previously for ASFV would be reflected by greater intra-sample genome-wide variation and elevated genomic polymorphisms between the wild-type isolates studied here. Our data suggest homogeneity of within-host and inter-genome sequences. These data are consistent with other studies describing factors supporting DNA fidelity in ASFV genome replication, including the activity of the viral class II apurinic/apyrimidinic (AP) endonuclease (E296R protein) [46] and the recently reported reparative role for Pol X in vivo [47, 48].

## Conclusions

These data provide insight into the intra-epidemic microevolution of ASFV and provides an early baseline for additional epidemiological tracing efforts in the region and elsewhere. While our effort was performed with limited resource availability in Tbilisi, Georgia, genomic data obtained directly from porcine blood was sufficient to yield genome-level data. Our results confirm the utility of deep sequencing as a highly informative culture-free method for ASFV genomic interrogation directly from PCR-positive blood. Future efforts may utilize additional sample processing methods to increase viral DNA yield prior to library preparation. Additional genome data from this epidemic will better facilitate targeted assay development and provide more robust epidemiological tracing as this epidemic continues.

## Additional file

Additional file 1: **Figure S1.** Genetic architecture of genomic region containing hypervariable G/C-tract loci, C315R/C147L locus repeat array structures, and BA71/BA71V CVR (pB602L) locus diversity. A) Location and organization of the six hypervariable G/C-tract loci. Box B) and C) illustrate examples of within-sample sequence read diversity at hypervariable G/C-tract loci with the MGF 110-14 L gene (B) and ASFV_G_ACD_00290 (C). Direct and indirect repeat structure of Georgia 2008/1 (D) and Georgia 2008/2 (E) calculated using DNASTAR GeneGuest repeat analysis. (F) Nucleic acid sequence alignment of CVR (pB602L) locus of BA71, BA71V and Por63. (PDF 15068 kb)

## Abbreviations

AP: apurinic/apyrimidinic
ASFV: African swine fever virus
BER: base excision repair
CVR: central variable region
DNA: Deoxyribonucleic acid
MGF: multi-gene family
PCR: Polymerase chain reaction
SARs: scaffold-associated regions
SNP: Single –nucleotide polymorphism
TRSs: tandem-repeat sequences
