# Supplementary figures and images for: Intra-epidemic genome variation in highly pathogenic African swine fever virus (ASFV) from the country of Georgia
Source: Virol J. 2018 Dec 14;15:190. doi: 10.1186/s12985-018-1099-z (PMC6295034; doi:10.1186/s12985-018-1099-z)

Figure S1

a)

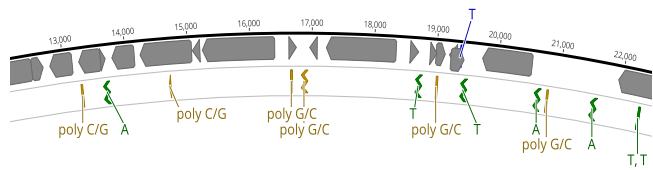

b)

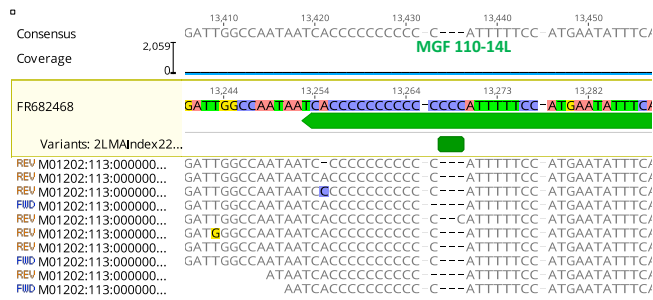

c)

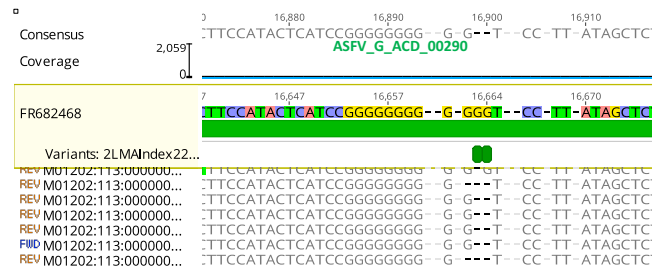

d)

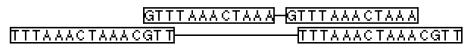

e)

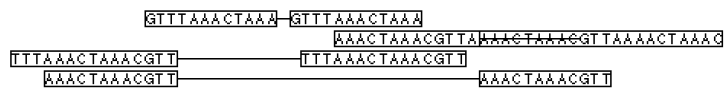

f)

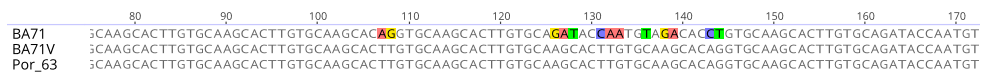

Supplement: Supplementary file 1 — Figure S1. Genetic architecture of genomic region containing hypervariable G/C-tract loci, C315R/C147L locus repeat array structures, and BA71/BA71V CVR (pB602L) locus diversity. A) Location and organization of the six hypervariable G/C-tract loci. Box B) and C) illustrate examples of within-sample sequence read diversity at hypervariable G/C-tract loci with the MGF 110-14 L gene (B) and ASFV_G_ACD_00290 (C). Direct and indirect repeat structure of Georgia 2008/1 (D) and Georgia 2008/2 (E) calculated using DNASTAR GeneGuest repeat analysis. (F) Nucleic acid sequence alignment of CVR (pB602L) locus of BA71, BA71V and Por63. (PDF 15068 kb) [file 12985_2018_1099_MOESM1_ESM.pdf]
